# Sleeping Problems or Emerging Psychosis? A Review of Emerging Literature

**DOI:** 10.1192/j.eurpsy.2024.1604

**Published:** 2024-08-27

**Authors:** J. Kim, G. Gill, S. Prasad, N. Roshan, B. Hasan, S. Gunturu

**Affiliations:** ^1^Psychiatry; ^2^Bronxcare Health System, Bronx, United States

## Abstract

**Introduction:**

Sleep disturbance, particularly insomnia, is prevalent across various mental health disorders. While it is a common sign in mood disorders, emerging evidence suggests that insomnia might act as a precursor or an early sign of psychosis. Our case report and literature review emphasize the importance of evaluating sleep disturbances in the diagnosis and management of mental disorders.

**Objectives:**

- To explore potential neurobiological underpinnings linking sleep disturbances to psychosis onset.
- To advocate for the importance of early identification and intervention for sleep disturbances in the broader context of preventing or managing psychotic disorders.

**Methods:**

We present a case describing a young patient’s first episode of psychosis, which was masked by an initial presentation of insomnia. Additionally, we conducted a review of the relationship between sleep disturbances and psychosis, with a comprehensive literature search from Pubmed, Scopus and psychINFO.

**Results:**

A 20-year-old African-American male with a history of poor sleep was initially diagnosed with Major Depressive Disorder. He was treated with Bupropion, Quetiapine, and Trazodone. However, he later presented with worsening depression, odd behavior, and signs of disorganization, suggestive of a psychotic episode. After switching his medication to Risperidone 4mg twice daily, the patient’s sleep and other symptoms markedly improved. Through our literature review, we identified that sleep disturbances, especially insomnia, can be a risk factor for developing psychosis. While a cross-sectional study recorded one-fourth of their study population experiencing First Episode Psychosis (FEP) with clinical insomnia, another study reported close to 80% of their study sample with early psychosis suffering from a minimum of one sleep disorder; insomnia and nightmare disorder being the most frequent. A large sample longitudinal analysis lasting one year also observed patients with sleep disorders to be twice at risk of onset and persistence of psychotic episodes. A growing body of evidence also suggests that structural brain abnormalities and neural development alterations in the early stages of psychosis may lead to sleep disturbances and subsequent psychotic symptoms. Findings suggest that thalamic dysfunction may in particular contribute to sleep spindle deficits and altered EEG microstate dynamics. These deficits are unrelated to antipsychotic medication exposure, and are also not observed in patients with other psychiatric illnesses.

**Conclusions:**

While the correlation between sleep disorders and psychosis has been well-established for decades, very limited literature is available on the role of sleep in FEP. Recognizing and treating sleep disturbances is pivotal in managing psychiatric disorders, including psychosis. Thus, a comprehensive evaluation of sleep issues in patients presenting with psychiatric symptoms is imperative for accurate diagnosis and management.

**Disclosure of Interest:**

None Declared

